# Comparison of efficacy and safety of traditional Chinese patent medicine in the treatment of vitiligo in children or adults: A protocol for systematic review and network meta-analysis

**DOI:** 10.1097/MD.0000000000024878

**Published:** 2021-03-19

**Authors:** Meng Yang, Mengmeng Du, Zhikun Tang, Guanqing Han, Wenyao Dong, Zhaoyu Chen, Yeqiang Song

**Affiliations:** a First College of Clinical Medicine, Shandong University of Traditional Chinese Medicine; b Affiliated Hospital of Shandong University of Traditional Chinese Medicine, Jinan, Shandong Province, China.

**Keywords:** network meta-analysis, protocol, traditional Chinese patent medicine, vitiligo in children or adults

## Abstract

**Background:**

Vitiligo is a common depigmented skin disease in children or adults, which usually causes considerable psychological burden to life and work for the reason that it affects appearance. The conventional therapies, including external 308 nm excimer laser therapy along with oral administration of western medicine, are associated with distinct disadvantages. Notably, traditional Chinese patent medicine (TCPM) exerts a vital part in treating vitiligo. Currently, no existing research has examined the effectiveness and safety of different TCPMs in treating vitiligo among either child or adult patients. As a result, the present network meta-analysis was carried out for the systematic comparison of the effectiveness of different TCPMs in treating vitiligo.

**Methods:**

The electronic databases, like PubMed, Web of Science, EMBASE, The Cochrane Library, Chinese Scientific Journals Database, China National Knowledge Infrastructure, Wanfang database and China BioMedical Literature, were searched systemically by 2 reviewers independently from inception to August 2020 to identify relevant randomized controlled trial (RCTs) according to our study inclusion criteria. In data extraction, risk of bias among those enrolled articles was also detected. Besides, the Bayesian network meta-analysis method was utilized to evaluate the evidence and data collected. This adopted the STATA and Win BUGS software for analysis.

**Results:**

The present work assessed the safety and efficacy of different TCPMs in treating vitiligo among child or adult patients.

**Conclusion:**

Our findings can shed precious lights on applying TCPMs in clinic and help the clinicians to formulate the efficient diagnostic and therapeutic strategies.

**Ethics and dissemination:**

No ethical approval was needed in this study.

**INPLASY registration number:**

INPLASY2020120050.

## 1 Introduction

Vitiligo refers to the frequently seen depigmentation disorder of skin resulting from the declined or lost functional melanocytes in the skin and/or hair follicles. The typical manifestation of skin lesions is depigmentation spots of varying size and number, and the prevalence rate is approximately 1%.^[1]^

In particular, the skin lesions of exposed areas such as face and neck seriously damage the appearance of patients. Patients with vitiligo usually have depression and anxiety, and this may result in social isolation or the sense of inferiority.^[2]^ According to a survey of its members conducted by the British Vitiligo Association, 57% of respondents said vitiligo exerted a moderate or severe impact on their quality of life.^[3]^ According to the 2012 consensus meeting on global issues of vitiligo and expert discussion, vitiligo can be divided into segmental type, vulgar type, mixed type and undetermined type of vitiligo.^[4]^① Segmental type: unilateral asymmetric vitiligo distributed along a cutaneous nerve segment (completely or partially matched to the skin segment), and a small number of bilateral multi-segmental vitiligo can be distributed. ② Nonsegmental (ordinary) type: including sporadic type, generalized type, facial neck type, limb type and mucous type; sporadic leukoplakia ≥2 pieces, area 1 to 3 grade; generalized leukoplakia area 4 grade (>50%); facial neck type, limb type and mucous type can all develop into generalized type. Mixed type: the coexistence of segmental type and nonsegmental type within 1 to 2 years. Undetermined type (original localized type): refers to a single skin lesion, the area is grade 1 and the fashion of medical treatment can not be determined as segmental or nonsegmental type. At present, there are the following drugs and methods for the treatment of vitiligo: glucocorticoids, calcineurin inhibitors (tacrolimus ointment, pimecrolimus cream. The instructions do not include the treatment of vitiligo, but it has been proved that these drugs are effective for vitiligo), vitamin D3 derivatives (carpotriol, tacasil), lattice laser therapy, skin grinding and transplantation therapy. The histopathology of vitiligo is characterized by the destruction of melanocytes in the local lesions and the decrease or disappearance of melanocytes. Based on modern medicine, the mechanism of melanocyte destruction in vitiligo is mainly associated with heredity, immunity, melanin self-destruction, spirit and nerve, oxidative stress and so on. At present, most scholars believe that vitiligo is a polygenic genetic disease. large-scale epidemiological investigations show that most cases of vitiligo are sporadic while about 10% of 36% of vitiligo patients tend to gather in families.^[5]^ According to the autoimmune hypothesis, chemokines and cytokines can recruit CD8+T cells, which finally accelerates epidermal melanocyte destruction.^[6]^ At present, a consensus is reached that stress exposure, cellular environment changes, toxic compound accumulation, melanocyte migration impairment and infection spread, may result in vitiligo.^[7,8]^ In addition, triggers such as skin trauma, hormonal changes and psychological stress in some women after pregnancy can also affect the progression of the disease.^[9]^ However, an increasing number of scholars believe that the imbalance of oxidative stress (Oxidative stress, OS) may be the initial factor in the pathogenesis of vitiligo. Excessive production of ROS in local epidermal microenvironment causes oxidative stress response, which leads to exposure of hidden antigenic epitope peptides in melanocytes, changes the immunogenicity of melanosome proteins, destroys the immune balance of the body as well as causes immune damage.^[10]^ Compared with other epidermal cells, melanocytes are particularly prone to oxidative stress. This is because melanocytes not only produce ROS when they are also stimulated by exogenous factors such as ultraviolet rays, but also produce a large amount of ROS in their unique melanin synthesis function.^[11]^ The degree of DNA oxidative damage of melanocytes under UV-induced oxidative stress was higher. Meanwhile, it was also found that the levels of antioxidant molecules SOD, GPx, and CAT in cultured melanocytes were lower than those in keratinocytes and fibroblasts.^[12,13]^

As the etiology and pathogenesis of vitiligo have not been fully clarified, the clinical efficacy of western medicine in the above classification is sometimes not satisfactory. As a result, more studies should be performed to examine the pathogenic mechanism of vitiligo. It has been verified in numerous systemic reviews and clinical trials that, traditional Chinese patent medicine (TCPMs) are effective on treating vitiligo. Traditional Chinese patent medicines for the treatment of vitiligo include Bailing pill, Qubaibabusi pill, Baishi capsule and Fu fang quchongbanjiu capsule. Basic research proves that Bailing pill is a traditional Chinese medicine preparation, which is considered by traditional Chinese medicine to exert the effect of nourishing blood and activating blood, removing blood stasis and dispelling wind. Western medicine believes that it can regulate immunity and increase photosensitivity, which is often used in the treatment of vitiligo. The main component of Qubaibabusi pill is psoralen extracted from Psoralea corylifolia, which is a traditional Chinese medicine. Modern pharmacological studies have shown that psoralen can increase the activity of tyrosinase, increase the production of melanocytes as well as further improve the level of melanin in the skin to achieve the effect of treating vitiligo.^[14]^ Through regulating the pharmacological effects of immune function, regulating the activity of tyrosinase, improving the activity of melanocytes, replenishing the content of local trace elements and so on, the synthesis of melanin by skin cells is promoted and vitiligo is treated.^[15]^ Network meta-analysis (NMA) allows for comparing the merits and demerits of at least 3 treatments, which takes full advantage of clinical data relative to the traditional meta-analysis. Consequently, NMA was adopted in this work for comparing the safety and efficacy of different TCPMs in the treatment of vitiligo among child or adult patients.

## 2 Methods

The Bayesian NMA method was adopted in the present work conducted following the PRISMA-P guidelines.

### 2.1 Study registration

The present NMA was registered at the International Platform of Registered Systematic Review and Meta-analysis Protocols (INPLASY registration number: INPLASY2020120050. Registration No.: URL= https://inplasy.com/inplasy-2020-12-0050/. DOI number: 10.37766/inplasy2020.12.0050).

### 2.2 Inclusion criteria

#### 2.2.1 Type of study

The eligible RCTs that adopted TCPMs to treat vitiligo among child or adult patients, together with relevant clinical trials (stage I/II/III trials, retrospective or prospective observation studies) were enrolled. In this study, only articles published in English or Chinese were adopted.

#### 2.2.2 Participants

Child or adult patients with vitiligo were enrolled. Anxiety was diagnosed following Vitiligo disease activity score (VIDA), clinical features, homomorphic reaction, Wood lamp examination results, laser confocal scanning microscope (CT) and dermatoscope image changes.

#### 2.2.3 Interventions

Patients in experimental group were given TCPMs in combination with traditional western medicine (WM). In the present study, the TCPMs adopted were Bailing pill, qubaibabusi pill, baishi capsule and fufangquchongbanjiuju capsule. WM treatment alone was applied for control group, including oral western medicine and external 308 nm excimer laser therapy. RCTs adopting at least 2 proprietary TCPMs or those applied moxibustion, acupuncture, as well as additional TCM treatments in combination were eliminated.

#### 2.2.4 Outcomes

According to the Vitiligo disease activity score (VIDA), clinical features, homomorphic reaction, Wood lamp examination results, laser confocal scanning microscope (CT) and dermatoscope image changes, all the enrolled articles should mention at least 1 main indicators.

### 2.3 Database and search strategy

Electronic databases, such as PubMed, Cochrane Library, Clinical Trials, Embase, VIP, China National Knowledge Infrastructure Database, China Biomedical Database, and Wanfang Database, were searched to identify eligible articles from inception to September 2020. The Medical Subject Headings (MeSH) were used in conjunction with keywords in the search strategy, which included “Traditional Chinese patent medicine, TCPM, Vitiligo in children or adults, Hypomelanosis, Hypomelanoses, Randomized controlled,” etc. (Table 1 lists the PubMed database search protocol.)

**Table 1:** Detailed search strategy for PubMed.

No.	Search item
#1	“vitiligo” [Mesh]
#2	(“Hypomelanoses” [Title/Abstract]) OR “Hypomelanosis” [Title/Abstract]
#3	children [Mesh]
#4	adults [Mesh]
#5	((Youth [Title/Abstract]) OR (Teenager [Title/Abstract])) OR (Teen [Title/Abstract])
#6	#1 OR #2 OR #3 OR #4 OR #5
#7	complementary therapies [MeSH Terms]
#8	complementary medicine [Title/Abstract] OR alternative therapies [Title/Abstract] OR medicine, alternative [Title/Abstract] OR Chinese patent medicine [Title/ Abstract] OR Chinese proprietary medicin [Title/Abstract] OR Chinese herbal drugs [Title/Abstract] OR herbal [Title/Abstract]
#9	Bailing pill [Title/Abstract] OR Qubaibabusi pill [Title/Abstract] OR Baishi capsule [Title/Abstract] OR Fu fang quchongbanjiu capsule [Title/Abstract]
#10	#7 OR #8 OR #9
#11	(Randomized controlled trial) [Publication Type] OR (Controlled clinical trial [Publication Type])
#12	(Randomized [Title/Abstract]) OR (random allocation [Title/Abstract])
#13	#11 OR #12
#14	#6 AND #10 AND #13

### 2.4 Study selection and data extraction

Two reviewers independently searched the databases following the study inclusion and exclusion criteria. Any disagreement between them was resolved by mutual discussion and negotiation with a third reviewer. The following information was collected, including ① basic data of all enrolled studies (title, first author, publication year, sample size, age, disease course, treatment duration); ② basic features of subjects and the related interventional measures; ③ critical elements in the assessment of risk of bias; ④ indicators of outcome.

### 2.5 Risk of bias assessment

Two reviewers evaluated the trial quality independently in line with the Cochrane Risk of Bias Risk Assessment Tool mentioned in the Cochrane Handbook version 5.1.0. In addition, the trial quality was assessed using the decision words such as “low risk,” “high risk,” or “unclear risk” from 7 perspectives, namely, blinding; sufficiency of random sequence; allocation concealment; selective reporting; complete result information; bias of publication; and others.

### 2.6 Statistical analysis

The Markov chain-Monte Carlo (MCMC) approach was adopted in this study for Bayesian meta-analysis using the Stata 14.0 software. Altogether 3 Markov chains were utilized in simulation for 50,000 iterations (the initial 20,000 were carried out for annealing and eliminating the original value impact, whereas the final 30,000 were adopted in sampling). We employed Stata 15.0 to draw the reticular diagram for directly and indirectly comparing diverse interventional measures. Relative odds ratio (ROR), together with the corresponding 95% confidence intervals (CIs), was determined for evaluating the closed loop consistency. As for 95% CI, its lower limit was 1, which indicated high consistency. Besides, a ROR value approximating 1 suggested the consistency between indirect and direct evidence; in this case, a fixed effect model was used. On the contrary, a random effect model was adopted due to the distinct inconsistency. Dichotomous data were presented as odds ratio together with the corresponding 95% CI. A difference of *P* < .05 indicated statistical significance. The effectiveness of diverse interventional measures was ranked using Win BUGS 1.4.3. The area under the curve (AUC), which was presented in the manner of percentage, was determined, with a higher value indicating a superior effect.

### 2.7 Assessment of heterogeneity

The fixed-effect model was adopted when *P* > .1 and *I*^2^ < 50%; or else, the possible heterogeneity source was explored and the random-effects model was used when there was no source detected.

### 2.8 Subgroup analysis and sensitivity analysis

In the presence of enough data, subgroup analysis was conducted. In addition, we also performed sensitivity analysis based on the symptom improvement rate for evaluating the methodology and clinical similarity among the enrolled articles, thus determining whether our results were creditable.

### 2.9 Evaluation of publication bias

The posttreatment total effective rate, Vitiligo disease activity score (VIDA), clinical features, homomorphic reaction, Wood lamp examination results, laser confocal scanning microscope (CT) and dermatoscope image changes were used to be the indicators. In addition, we plotted an inverted funnel plot, where the effect amount was used to be horizontal coordinate, while its standard error was used to be the vertical coordinate. In the present work, the symmetry of this inverted funnel plot indicated the presence of small sample effect or the low likelihood of bias of publication.

### 2.10 Grading the quality of evidence

GRADE was adopted for evaluating the evidence quality in 5 perspectives shown below: indirectness, risk of bias, imprecision, inconsistency, together with bias of publication.^[16]^

## 3 Discussion

Due to the difficulty in the treatment of vitiligo and repeated conditions, there is no good treatment so far, such as drug treatment, skin grafting, decolorization therapy and physiotherapy, which can not achieve the purpose of cure. However, with the development of modern medical technology, as well as modern doctors’ exploration and innovation of traditional Chinese patent medicine, there has been some experience and curative effect in the treatment of vitiligo. For localized and stable patients, surgical operation, traditional Chinese patent medicine and acupuncture treatment can be used. For patients with extensive type, acute stage, progressive stage and severe skin lesions, internal medication and physiotherapy should be used. Do not rigidly adhere to the means of treatment, the use of integrated traditional Chinese and western medicine treatment plan, each should take their strengths in order to improve the clinical effect. Traditional Chinese patent medicine was highly compatible based on the TCM theory supervised by related national departments, which has demonstrated its effectiveness when it is used for a long time in clinic. Currently, no existing study has compared the merits and demerits of different TCPMs in treating vitiligo. Therefore, it is of great necessity to adopt the NMA approach to investigate such topic. The NMA approach was introduced in the present work based on existing RCTs for the sake of evaluating the merits and demerits of different TCMPs, which might offer an efficient diagnostic and therapeutic strategy for clinicians.

## Author contributions

**Conceptualization:** Meng Yang, Yeqiang Song.

**Data curation:** Meng Yang, Mengmeng Du.

**Formal analysis:** Meng Yang, Zhaoyu Chen, Zhikun Tang.

**Funding acquisition:** Yeqiang Song.

**Investigation:** Meng Yang.

**Methodology:** Meng Yang, Mengmeng Du, Guanqing Han.

**Project administration:** Meng Yang.

**Resources:** Meng Yang.

**Software:** Meng Yang, Mengmeng Du, Wenyao Dong.

**Validation:** Meng Yang.

**Visualization:** Meng Yang.

**Writing – original draft:** Meng Yang, Mengmeng Du.

**Writing – review & editing:** Meng Yang, Yeqiang Song
